# Mitochondrial Genome Size and D-Loop Variation in Flying, Aquatic, and Terrestrial Mammals: An Exploratory Comparative Analysis

**DOI:** 10.3390/genes17101294

**Published:** 2026-10-16

**Authors:** Haiquan Zhang, Feiyun Tu, Wenxuan Liu, Yang Yang

**Affiliations:** Key Laboratory of Tropical Animal and Plant Ecology of Hainan Province, Ministry of Education Key Laboratory for Ecology of Tropical Islands, College of Life Sciences, Hainan Normal University, Haikou 571158, China; 202208071238@hainnu.edu.cn (H.Z.); 201908071510@hainnu.edu.cn (W.L.); yy619@hainnu.edu.cn (Y.Y.)

**Keywords:** mammals, mitochondrial genome, *D-loop*, ecological adaptation, energy metabolism, phylogenetic non-independence, PGLS

## Abstract

**Background:** Mitochondrial genome size varies widely among mammals, yet whether this variation is systematically associated with major ecological transitions in locomotor mode remains unclear. Here, we compared complete mitochondrial genome length and the lengths of the *D-loop*, *12S rRNA*, and *16S rRNA* among flying, aquatic, and terrestrial mammals. **Methods:** We used 13,487 complete mitogenome sequences retrieved from NCBI, representing 1674 species across 22 mammalian orders. Non-parametric tests and phylogenetic generalized least squares (PGLS) analyses were used to assess differences and associations. **Results:** Non-parametric tests showed that all four genomic features differed significantly among ecological groups (Kruskal–Wallis, all *p* < 0.001). Complete genome length and *D-loop* length showed concordant patterns (flying > terrestrial > aquatic), whereas rRNA lengths followed distinct patterns, with aquatic mammals possessing the longest *12S* and *16S rRNA* genes. However, the absolute median differences in rRNA lengths were small (18 bp for *12S rRNA* and 12 bp for *16S rRNA*), and the *12S rRNA* effect size was not negligible (ϵ^2^ = 0.22), so these differences should not be interpreted as evidence of adaptive divergence. Phylogenetic generalized least squares (PGLS) analyses further showed that ecological group was not significantly associated with any genomic feature after accounting for phylogenetic non-independence (all overall *p* > 0.10)—a conservative result that, given the very small number of independent ecological transitions, reflects limited statistical power as much as the absence of ecological effects. Under the taxonomic proxy tree, appreciable phylogenetic structure was detected for *D-loop* length (Pagel’s λ = 0.51) and complete genome size (λ = 0.74), but these values should not be interpreted as strict measures of phylogenetic signal. **Conclusions:** Because powered flight evolved only once in mammals (Chiroptera) and the fully aquatic lifestyle only twice (Cetacea and Sirenia), these group-level patterns rest on few independent evolutionary transitions and cannot be statistically separated from lineage-specific or ancestral states. We therefore present these findings as an exploratory, hypothesis-generating comparison. Phylogenetically explicit analyses based on a fully resolved species tree, structural characterization of control-region variation, and direct functional assays of replication and transcription efficiency will be required to test the adaptive scenarios proposed here.

## 1. Introduction

In 2005, Science published 125 major scientific questions, one of which posed a fundamental problem in genomics: “Why are some genomes really big and others quite compact?” [1]. Genome size variation is often strongly influenced by differences in non-coding regions [2]. Although this phenomenon is most commonly discussed in the context of large nuclear genomes, non-coding-region-associated variation is also evident in the much more compact mitochondrial genome.

Mitochondria are the primary energy-producing organelles of eukaryotic cells, generating most cellular ATP through oxidative phosphorylation. As semi-autonomous organelles, their function depends on coordinated interactions between the mitochondrial and nuclear genomes. Oxidative phosphorylation supplies approximately 95% of the ATP required for locomotor activity, and the enzyme complexes involved are composed of subunits encoded by both genomes [3,4]. The mitochondrial genome encodes core components of the respiratory chain and is therefore essential for cellular energy conversion. Compared with the large and structurally complex nuclear genome, the mitochondrial genome is compact, functionally conserved, and characterized by distinctive evolutionary dynamics, making it an important model in molecular evolution and comparative genomics.

Mitochondrial DNA (mtDNA) is typically a closed, circular, double-stranded molecule [5]. Most metazoan mitochondrial genomes are approximately 16 kb in length and encode 37 tightly arranged genes [6], including 13 protein-coding genes involved in oxidative phosphorylation, 22 transfer RNA genes, and two ribosomal RNA genes [5,6]. These genes constitute the core genetic machinery required for mitochondrial protein synthesis and energy production. In addition, mtDNA is characterized by maternal inheritance and a relatively rapid evolutionary rate [7,8], which have made it widely used in population genetics, phylogeography, and molecular phylogenetics [8,9].

Animal mitochondrial genomes also contain a major non-coding region known as the control region or *D-loop* [5]. This region harbors key regulatory elements involved in mtDNA replication and transcription, including the LSP, HSP, TAS, and conserved sequence blocks (CSB1-3), as well as the origin of heavy-strand replication (OH) [10,11,12]. The *D-loop* is generally the most variable part of the mitochondrial genome in both sequence composition and length [5,10]. Interspecific differences in mitochondrial genome length are largely attributable to variation in this region, particularly through the gain or loss of tandem repeat sequences [10,13]. Consequently, the *D-loop* provides an important genomic feature for examining mitochondrial genome size variation and its potential evolutionary significance.

Mammals have diversified into a wide range of ecological environments, including terrestrial, aquatic, and aerial habitats. These ecological transitions are associated with major differences in locomotor mode, oxygen availability, thermoregulation, and energy metabolism. For example, aquatic mammals often experience hypoxia and oxidative stress during diving [14], whereas flying mammals must sustain the high energetic costs of powered flight [3]. Such differences in metabolic demand may impose distinct selective pressures on mitochondrial genomes [15]. Because mitochondria are central to ATP production, mitochondrial genome evolution is closely linked to species-specific metabolic requirements. Previous studies have demonstrated that functional constraints on mitochondrially encoded proteins differ among organisms with different metabolic rates, thereby influencing patterns of mitochondrial genome evolution [16]. Comparative analyses of mitochondrial protein-coding genes have revealed evidence of positive selection in both flying bats [3] and diving cetaceans [17,18], suggesting that ecological specialization drives adaptive evolution of energy metabolism genes. In addition, mitochondria are a major intracellular source of reactive oxygen species (ROS), and elevated ROS levels can affect mitochondrial genome stability through oxidative DNA damage, including base modifications and deletions [19].

In cetaceans, substantial length variation has been reported in the mitochondrial *D-loop*, whereas protein-coding genes generally show Ka/Ks ratios far below 1, indicating strong purifying selection [20]. Mitochondrial tRNAs in cetaceans also exhibit signatures of adaptive evolution [21]. These findings suggest that aquatic environments may have influenced multiple aspects of mitochondrial genome evolution. Similarly, studies in bats indicate that the evolutionary rate of mtDNA may differ from that of other mammals and that genes associated with energy metabolism may have undergone adaptive evolution in response to the elevated energetic demands of flight [3,22].

Although the general characteristics of mammalian mitochondrial genomes have been extensively studied, and many studies have examined mitochondrial genome evolution in ecologically specialized groups [23,24,25], previous research has largely focused on protein-coding sequence variation for phylogenetic reconstruction or on specific taxa [15,26,27]. A unified comparative framework for systematically evaluating mitochondrial genome size and the lengths of its major components among aquatic, terrestrial, and flying mammals remains lacking. Furthermore, the relative contributions of the *D-loop* versus coding regions to genome size differences across ecological groups, and the potential mechanisms underlying these patterns, have not been comprehensively addressed.

Importantly, any comparison of flying, aquatic, and terrestrial mammals faces an intrinsic phylogenetic constraint: powered flight originated only once in mammals (in the lineage leading to Chiroptera), and the fully aquatic lifestyle was acquired independently only twice (in Cetacea and Sirenia). Consequently, species within each ecological group are not statistically independent observations, and group-level differences may reflect the idiosyncratic history of one or a few lineages rather than convergent responses to ecological pressures. We therefore frame the present study explicitly as an exploratory, descriptive comparison that documents large-scale patterns and generates hypotheses, rather than as a formal test of adaptive causation.

In this study, we downloaded mammalian mitochondrial genome sequences from the NCBI database and classified species into aquatic, terrestrial, and flying groups according to their primary ecological niche and locomotor mode. Using non-parametric statistical tests, we compared complete mitochondrial genome length and the lengths of major mitochondrial components, including the *D-loop*, *12S rRNA*, and *16S rRNA*, among these groups. Our specific objectives were: (i) to determine whether mitochondrial genome size and *D-loop* length differ significantly among flying, aquatic, and terrestrial mammals; (ii) to assess whether coding-region lengths (*12S* and *16S rRNA*) show comparable patterns of variation; and (iii) to discuss—with explicit caution regarding phylogenetic non-independence—the potential evolutionary mechanisms (including metabolic demand, selective pressures, and genetic drift) that may underlie the observed patterns. By linking mitochondrial genome size variation with broad ecological adaptations, this study provides a comparative perspective on mitochondrial genome evolution in mammals and contributes evidence relevant to the broader question of why some genomes are relatively large whereas others are highly compact.

## 2. Materials and Methods

### 2.1. Data Collection

Complete mitochondrial genome sequences were retrieved from the National Center for Biotechnology Information (NCBI) database using Python v3.8 scripts. When a mitochondrial genome was represented by both a RefSeq accession (NC_) and its original GenBank submission, only the NCBI-curated RefSeq sequence was retained; the corresponding original GenBank record was excluded to avoid duplicate sampling of the same genome. The taxonomic scope covered 22 mammalian orders following Burgin et al. [28]: Carnivora, Cetartiodactyla, Chiroptera, Eulipotyphla, Lagomorpha, Afrosoricida, Macroscelidea, Monotremata, Perissodactyla, Pilosa, Proboscidea, Rodentia, Scandentia, Sirenia, Dermoptera, Cingulata, Dasyuromorphia, Diprotodontia, Hyracoidea, Pholidota, Primates, and Tubulidentata (Table 1). To ensure data accuracy for subsequent analyses, we manually removed duplicate records derived from the same individual, as well as mitochondrial genome sequences that were annotated as complete but were not actually fully sequenced. The lengths of the *D-loop*, *12S rRNA*, and *16S rRNA* regions were extracted and calculated based on the annotated gene boundaries provided in the corresponding GenBank records for each mitochondrial genome (Table S1).

For each complete mitochondrial genome, the full circular sequence was retrieved. Because the control region is not consistently annotated across GenBank records, we operationally defined the *D-loop* as the non-coding interval between the *tRNA-Pro* and *tRNA-Phe* genes, which flanks the control region in the canonical mammalian mitogenome gene order. The *D-loop* length was therefore calculated as the number of bases between the 3′ end of *tRNA-Pro* and the 5′ end of *tRNA-Phe*. The boundaries of the *12S* and *16S* rRNA genes were taken directly from the corresponding GenBank feature annotations.

### 2.2. Data Processing

#### 2.2.1. Ecological Grouping

Mammals were classified into three ecological groups according to their primary locomotor mode and core ecological niche (Table 1): Group 1 (flying mammals), comprising one order and 196 species (n = 383); Group 2 (aquatic mammals), comprising two orders and 77 species (n = 1849); and Group 3 (terrestrial mammals), comprising 20 orders and 1401 species (n = 11,255).

We emphasize that this classification is based on the predominant locomotor environment of each order rather than on the aquatic specialization of individual species or families. Consequently, several semi-aquatic lineages—such as Hippopotamidae within Artiodactyla, Lutrinae and Pinnipedia within Carnivora, and Castoridae within Rodentia—are nested within the terrestrial group because their parent orders are primarily terrestrial. Similarly, gliding squirrels (Pteromyini) and arboreal primates remain within the terrestrial group because their orders lack powered flight. This taxonomic-level grouping is methodologically necessary to maintain non-overlapping categories for statistical comparison, but it inevitably introduces ecological heterogeneity into the terrestrial group.

#### 2.2.2. Statistical Comparisons of Mitochondrial Genome Features

Statistical analyses of complete mitochondrial genome length and the lengths of the *D-loop*, *12S rRNA*, and *16S rRNA* were performed using IBM SPSS Statistics v30.0. Shapiro–Wilk tests were first applied to assess normality; because all datasets deviated significantly from normality (*p* < 0.01), Kruskal–Wallis tests were used to evaluate differences among these three ecological groups. Dunn’s post hoc tests were subsequently applied for pairwise multiple comparisons. Effect sizes were quantified as epsilon-squared (ε^2^ = (H − k + 1)/(n − k)) for the Kruskal–Wallis tests. Epsilon-squared values range from 0 to 1; values of approximately 0.01, 0.06, and 0.14 are interpreted as small, medium, and large effect sizes [29], respectively.

#### 2.2.3. Robustness Checks and Species-Level Comparison

To validate the comparative results of complete mitochondrial genome length, as well as the lengths of the *D-loop*, 1*2S rRNA*, and *16S rRNA*, two complementary approaches were employed. First, given the substantial imbalance in sample size among the three ecological groups, random subsampling was conducted to enhance result robustness. Specifically, 383 records were randomly selected from both the aquatic and terrestrial mammal groups (each matching the sample size of the flying mammal group) for three independent iterations, and the aforementioned statistical analyses were repeated. These subsampling datasets are provided in Table S2. Second, to mitigate the confounding effect of intraspecific sequence redundancy, the median sequence length was calculated for each species represented by multiple entries, and this median value was subsequently used as the representative species-level estimate for all statistical comparisons. The median sequence lengths for complete mitochondrial genomes, *D-loop*, 1*2S rRNA*, and *16S rRNA* are also provided in Table S3.

#### 2.2.4. Exploratory Subgroup Analysis of Gliding Mammals

For the exploratory subgroup analysis of gliding mammals, gliding taxa were defined as those in the patagial-gliding lineages (Pteromyini and Petauridae), the scaly-tailed squirrels (Anomaluridae), and the colugos (Dermoptera), and were compared against all other terrestrial mammals using a two-sided Mann–Whitney U test.

### 2.3. Nucleotide Composition Analyses

Based on the GenBank accession numbers listed in Table S1, we downloaded 13,487 *D-loop* sequences. Because some species have multiple sequences, we randomly selected one *D-loop* sequence per species (Table S3). The nucleotide composition of each mitochondrial *D-loop* sequence was calculated using MEGA v11.0 [30]. For statistical comparisons, the A, T, C, and G percentages of each *D-loop* sequence were treated as species-level records without length weighting. Because the four nucleotide proportions are compositional and sum to 100%, each base proportion was analyzed separately as a marginal comparison. Differences among the three ecological groups were assessed using Kruskal–Wallis tests, followed by pairwise two-sided Mann–Whitney U tests with Bonferroni-adjusted *p* values.

### 2.4. Phylogenetic Generalized Least Squares Analyses

To explicitly address phylogenetic non-independence, we additionally performed phylogenetic generalized least squares (PGLS) with Pagel’s λ estimated by maximum likelihood, using species-level median values (n = 1674; Table S3) and a phylogenetic variance–covariance matrix derived from the nested taxonomic hierarchy (order, family, genus) as a proxy for the interspecific phylogeny. The response variables were *D-loop*, *12S rRNA*, *16S rRNA*, and complete genome length; the predictor was ecological group (flying/aquatic/terrestrial, with terrestrial as the reference level).

## 3. Results

### 3.1. Complete Mitochondrial Genome Length

**Figure 1:**
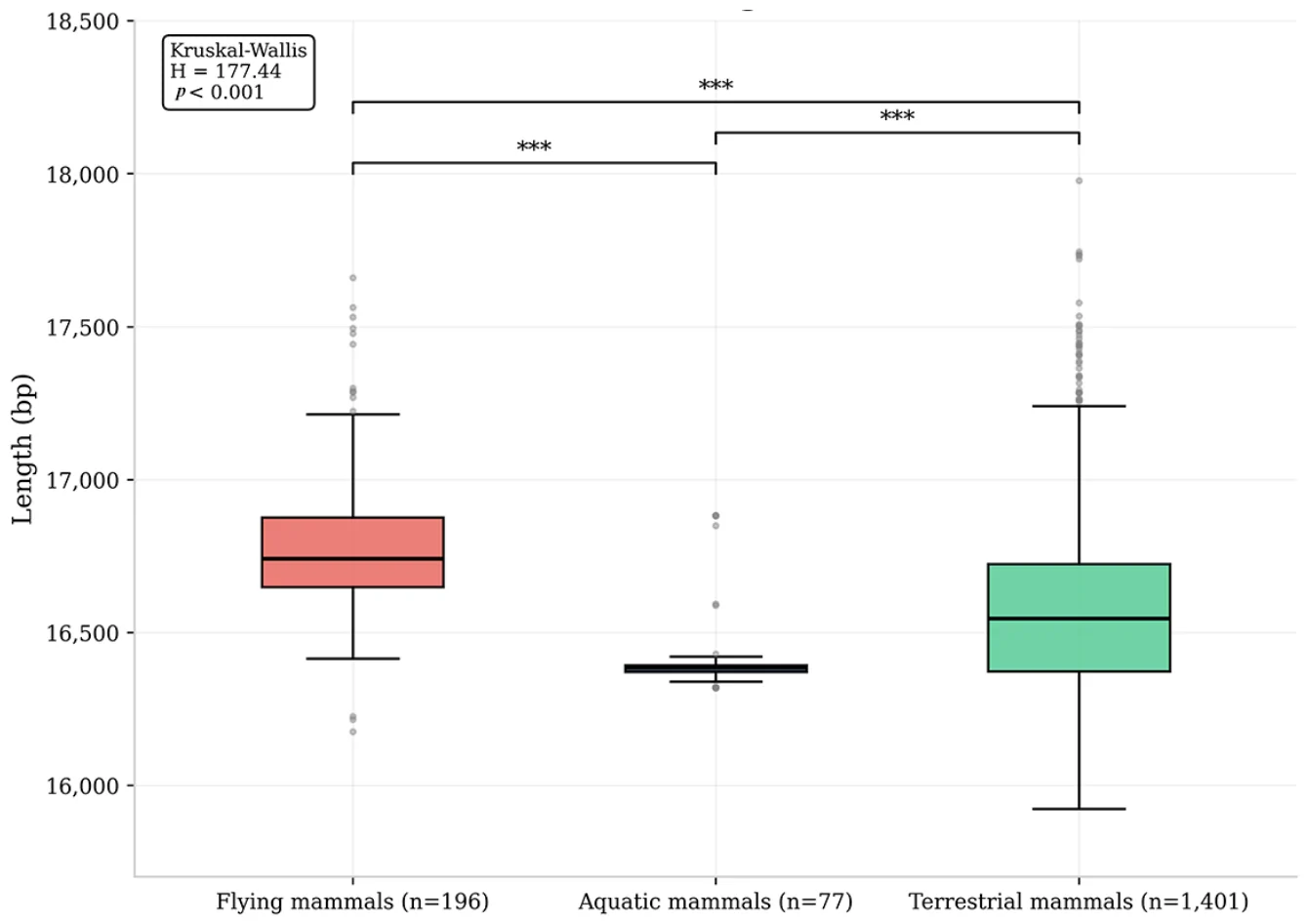
Kruskal–Wallis test for complete mitochondrial genome size (*** indicates *p* < 0.001).

To account for the large imbalance in record numbers and the non-independence of GenBank records (i.e., multiple records per species), we calculated the median mitochondrial genome length for each species and used these species-level values for the primary statistical analysis. Mitochondrial genome length differed significantly among the three ecological groups (Kruskal–Wallis test: H = 177.44, *p* < 0.001, ε^2^ = 0.10; Figure 1). The median mitochondrial genome length was 16,740 bp in flying mammals, 16,386 bp in aquatic mammals, and 16,544 bp in terrestrial mammals (Table S4). Dunn’s multiple comparisons showed that the median genome length was significantly greater in flying mammals than in aquatic mammals, significantly greater in flying mammals than in terrestrial mammals, and significantly greater in terrestrial mammals than in aquatic mammals (Figure 1). Thus, the overall pattern was flying mammals > terrestrial mammals > aquatic mammals. A record-level analysis of all GenBank records (n = 383 flying, 1849 aquatic, and 11,255 terrestrial records) yielded results consistent with the species-level analysis (Figure S1), and a random subsampling analysis produced the same ranking (Figure S2).

### 3.2. D-Loop Length

**Figure 2:**
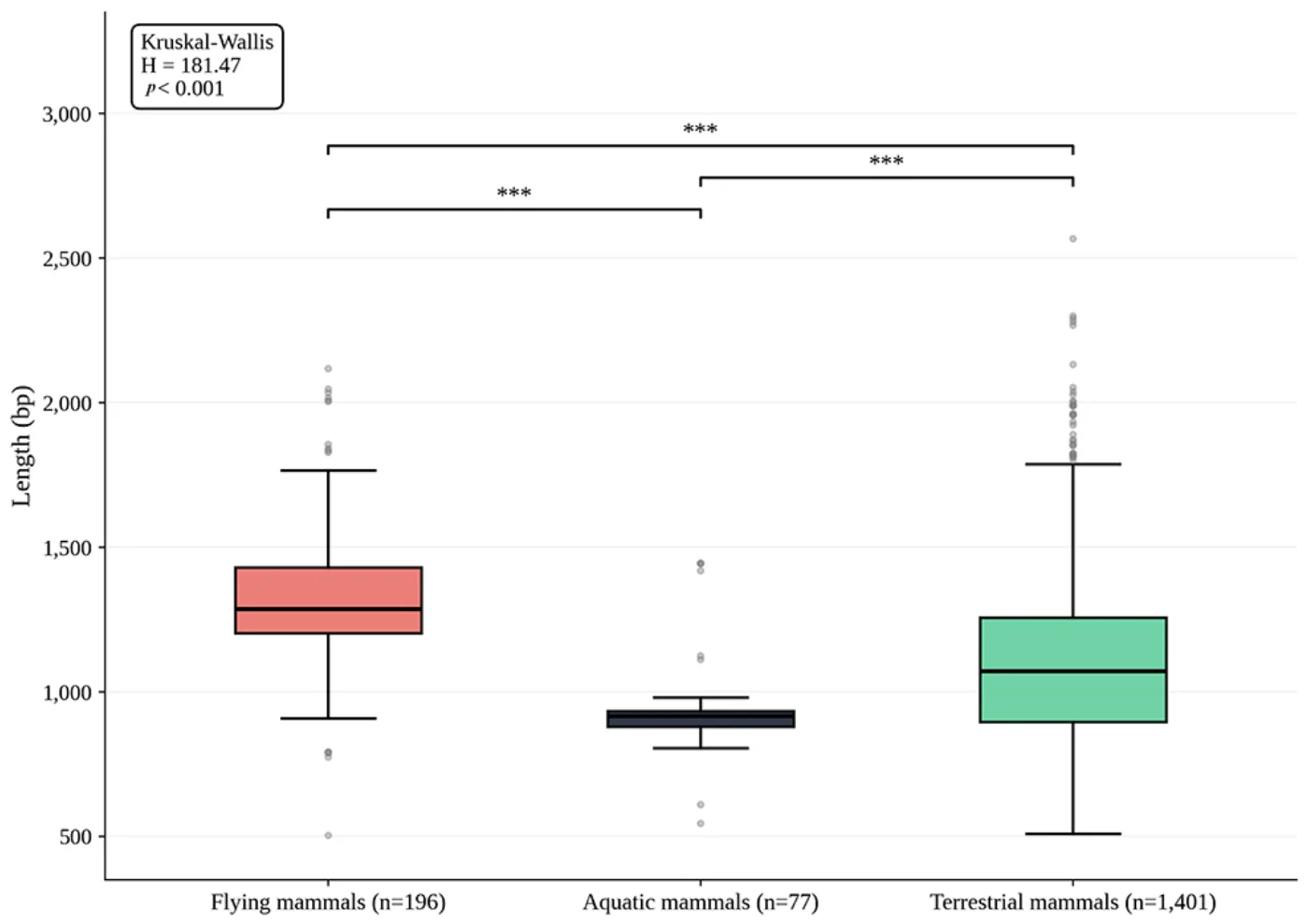
Kruskal–Wallis test for *D-loop* length (*** indicates *p* < 0.001).

The median length of the mitochondrial *D-loop* also differed significantly among the three ecological groups based on species-level values. Flying mammals had the longest *D-loop*, with a median length of 1284 bp, followed by terrestrial mammals (1069 bp) and aquatic mammals (914 bp) (Table S5). The Kruskal–Wallis test indicated a highly significant difference among groups (H = 181.47, *p* < 0.001, ε^2^ = 0.10; Figure 2). Dunn’s multiple comparisons showed that *D-loop* length was significantly greater in flying mammals than in terrestrial mammals, significantly greater in flying mammals than in aquatic mammals, and significantly greater in terrestrial mammals than in aquatic mammals (Figure 2). Therefore, the observed pattern was flying mammals > terrestrial mammals > aquatic mammals. A record-level analysis of all GenBank records (n = 383 flying, 1849 aquatic, and 11,255 terrestrial records) yielded results consistent with the species-level analysis (Figure S1), and a random subsampling analysis produced the same ranking (Figure S2).

### 3.3. Nucleotide Composition of the Mitochondrial D-Loop

The overall sequence-length-weighted nucleotide composition of the mitochondrial *D-loop* differed among the three ecological groups. Flying mammals had the highest A and G contents, aquatic mammals had the highest T content, and terrestrial mammals had the highest C content. The overall *D-loop* nucleotide composition was 32.3% A, 26.3% T, 26.0% C, and 15.4% G in flying mammals; 30.0% A, 30.7% T, 24.5% C, and 14.8% G in aquatic mammals; and 31.9% A, 28.8% T, 25.5% C, and 13.8% G in terrestrial mammals. At the sequence level, the mean A content was 32.10%, 29.91%, and 31.68%; the mean T content was 26.22%, 30.87%, and 28.88%; the mean C content was 26.07%, 24.36%, and 25.60%; and the mean G content was 15.61%, 14.86%, and 13.84% in flying, aquatic, and terrestrial mammals, respectively (Table 2).

The composition of all four nucleotides differed significantly among the ecological groups (A: Kruskal–Wallis H = 65.548, *p* = 5.84 × 10^−15^, ε^2^ = 0.038; T: H = 185.561, *p* = 5.08 × 10^−41^, ε^2^ = 0.110; C: H = 30.116, *p* = 2.89 × 10^−7^, ε^2^ = 0.017; G: H = 136.943, *p* = 1.83 × 10^−30^, ε^2^ = 0.081). T content showed the largest among-group effect, followed by G and A, whereas C showed the smallest effect size. For T and G, all pairwise ecological-group comparisons remained statistically significant after Bonferroni correction. For A and C, flying mammals differed significantly from aquatic mammals, and aquatic mammals differed significantly from terrestrial mammals; however, the difference between flying and terrestrial mammals was not significant after Bonferroni correction for either A (*p* = 0.147) or C (*p* = 0.149). Overall, A and C contents followed the pattern flying mammals > terrestrial mammals > aquatic mammals, T content followed the pattern aquatic mammals > terrestrial mammals > flying mammals, and G content followed the pattern flying mammals > aquatic mammals > terrestrial mammals.

### 3.4. 12S rRNA Length

**Figure 3:**
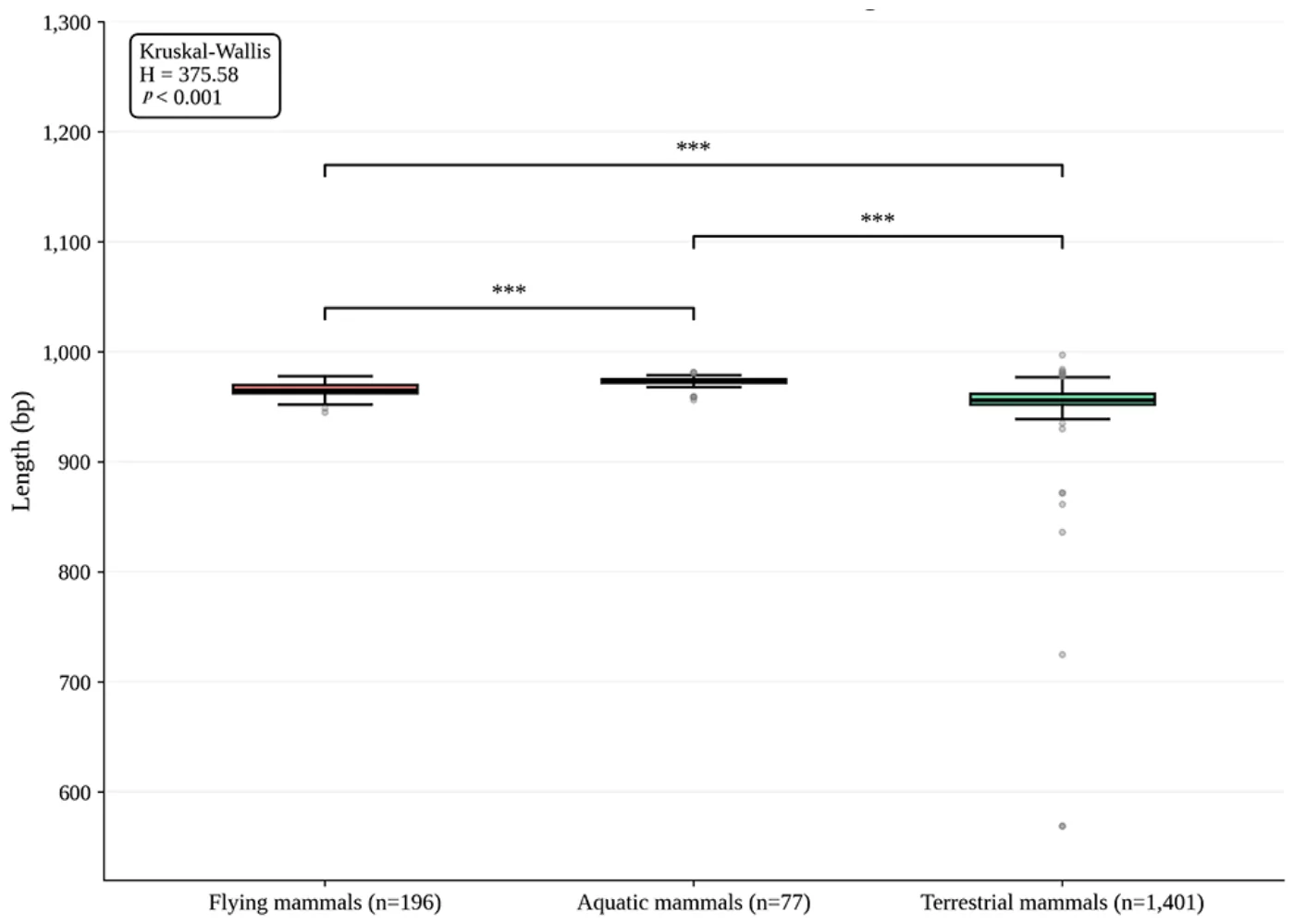
Kruskal–Wallis test for *12S rRNA* length (*** indicates *p* < 0.001).

The length of the mitochondrial *12S rRNA* gene also differed significantly among the three groups based on species-level medians (Kruskal–Wallis test: H = 375.58, *p* < 0.001, ε^2^ = 0.22; Figure 3). The median lengths were 965 bp in flying mammals, 974 bp in aquatic mammals, and 956 bp in terrestrial mammals (Table S6). Dunn’s multiple comparisons indicated that *12S rRNA* length was significantly greater in aquatic mammals than in flying mammals, significantly greater in aquatic mammals than in terrestrial mammals, and significantly greater in flying mammals than in terrestrial mammals (Figure 3). Thus, the observed pattern was aquatic mammals > flying mammals > terrestrial mammals. A record-level analysis of all GenBank records (n = 383 flying, 1849 aquatic, and 11,255 terrestrial records) yielded results consistent with the species-level analysis (Figure S1), and a random subsampling analysis produced the same ranking (Figure S2).

### 3.5. 16S rRNA Length

**Figure 4:**
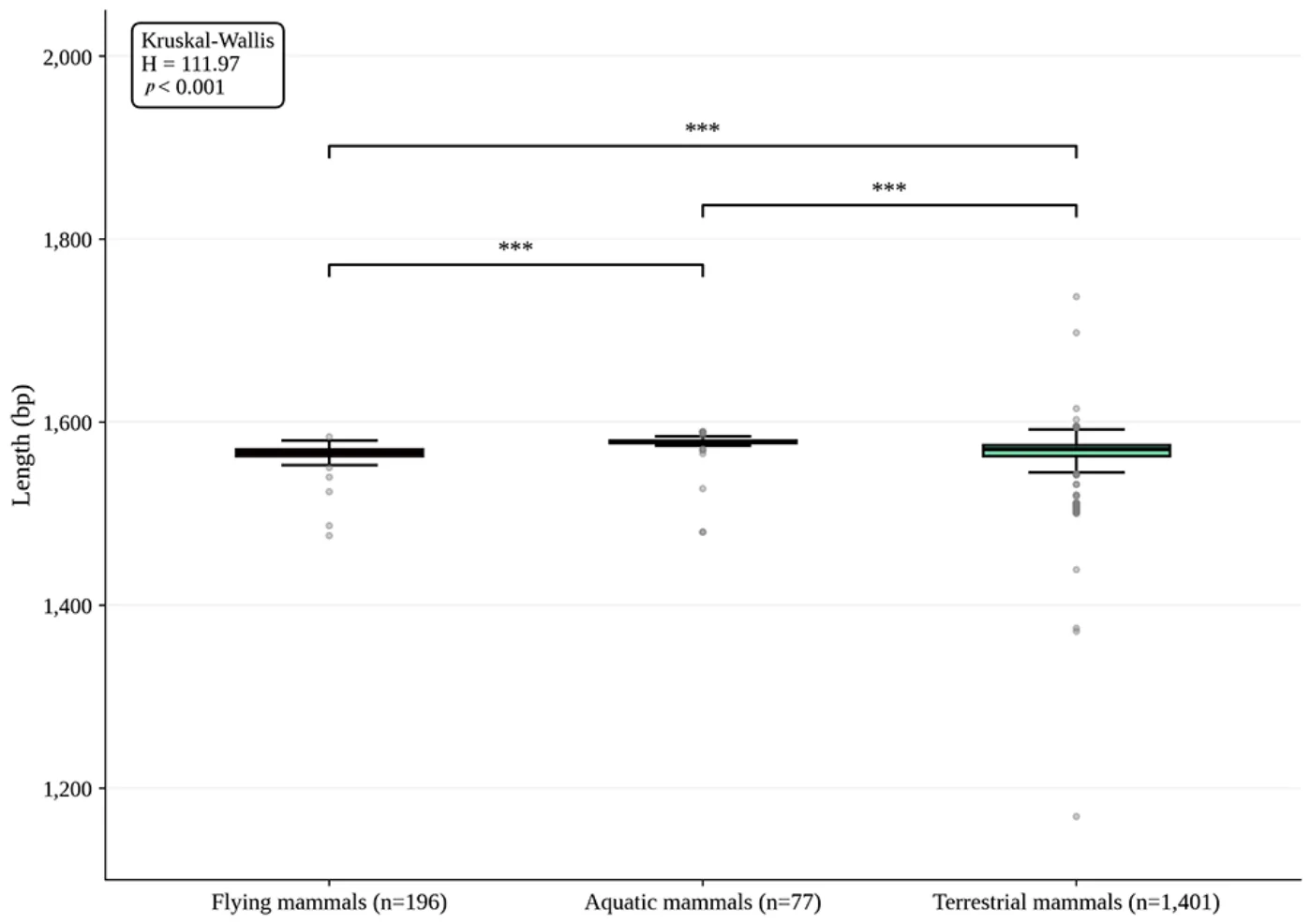
Kruskal–Wallis test for *16S rRNA* length (*** indicates *p* < 0.001).

The mitochondrial *16S rRNA* gene length differed significantly among the three groups based on species-level medians (Kruskal–Wallis test: H = 111.97, *p* < 0.001, ε^2^ = 0.07; Figure 4). The median lengths were 1566 bp in flying mammals, 1578 bp in aquatic mammals, and 1570 bp in terrestrial mammals (Table S7). Dunn’s multiple comparisons showed that *16S rRNA* length was significantly greater in aquatic mammals than in flying mammals, significantly greater in aquatic mammals than in terrestrial mammals, and significantly greater in terrestrial mammals than in flying mammals (Figure 4). The resulting pattern was aquatic mammals > terrestrial mammals > flying mammals. A record-level analysis of all GenBank records (n = 383 flying, 1849 aquatic, and 11,255 terrestrial records) yielded results consistent with the species-level analysis (Figure S1), and a random subsampling analysis produced the same ranking (Figure S2).

### 3.6. PGLS Analysis

The Kruskal–Wallis tests reported above address whether the distributions of each genomic feature differ among ecological groups, whereas the PGLS analyses test whether ecological group remains associated with each feature after accounting for shared evolutionary history; the two sets of results therefore address distinct questions and are not in direct contradiction. PGLS revealed appreciable phylogenetic structure for complete mitochondrial genome size (Pagel’s λ = 0.74) and *D-loop* length (λ = 0.51), and weaker structure for the rRNA genes (λ = 0.18 for both *12S* and *16S*). After phylogenetic correction, ecological group was not significantly associated with any of the four genomic features (Table 3). For the *D-loop*, the contrasts relative to terrestrial mammals were −116.7 ± 101.9 bp for aquatic mammals (*p* = 0.252) and 44.9 ± 119.0 bp for flying mammals (*p* = 0.706); the overall likelihood-ratio test was non-significant (χ^2^ = 1.5, *p* = 0.463). For *12S rRNA*, the aquatic contrast was 11.8 ± 6.2 bp (*p* = 0.059), representing only a marginal positive trend that did not survive correction for multiple testing; the overall test was likewise non-significant (χ^2^ = 4.5, *p* = 0.107). For *16S rRNA*, neither the aquatic contrast (3.7 ± 6.6 bp, *p* = 0.568) nor the flight contrast (−4.1 ± 7.3 bp, *p* = 0.574) approached significance (overall *p* = 0.703). Complete genome size showed the same pattern (aquatic: −107.8 ± 104.6 bp, *p* = 0.303; flight: 49.6 ± 123.4 bp, *p* = 0.688; overall *p* = 0.521).

## 4. Discussion

### 4.1. D-Loop Length Variation

The present study reveals a striking concordance between complete mitochondrial genome length and *D-loop* length across flying, aquatic, and terrestrial mammals: flying mammals possess the longest mitochondrial genomes and *D-loop* regions, terrestrial mammals are intermediate, and aquatic mammals have the shortest. This parallel pattern is consistent with the hypothesis that variation in the *D-loop* region may be the principal determinant of differences in total mitochondrial genome size among ecological groups. We emphasize, however, that this concordance is correlational: it identifies the *D-loop* as the genomic compartment most likely to underlie the length differences, but it does not by itself establish why those differences evolved.

The mammalian *D-loop* is a structurally complex non-coding region that serves as the primary regulatory hub for mtDNA replication and transcription [10,11,12]. It contains essential functional elements, including the heavy-strand and light-strand promoters (HSP and LSP), three conserved sequence blocks (CSB1-3) involved in RNA primer processing for heavy-strand replication, termination-associated sequences (TASs) that regulate *D-loop* strand synthesis, and the origin of heavy-strand replication [10,11,12]. The substantial length variation observed in the *D-loop*, ranging from approximately 914 bp (median value) in aquatic mammals to 1284 bp (median value) in flying mammals, likely reflects differences in the abundance and length of tandem repeat sequences, which are known to be highly variable across species [10,13]. These tandem repeats are frequently located in the extended termination-associated sequence (ETAS) domain and the CSB domain and have been implicated in the regulation of mtDNA replication efficiency [11,31]. The finding that the *D-loop* accounts for the majority of genome size differences among ecological groups is consistent with the broader observation that coding regions of the mitochondrial genome are highly conserved, whereas non-coding regions tolerate greater structural flexibility [5,16]. In addition to the marked differences in *D-loop* length, the nucleotide composition of the *D-loop* also differed significantly among ecological groups for all four bases. The largest compositional difference was observed for T content (ε^2^ = 0.110), which was highest in aquatic mammals, intermediate in terrestrial mammals, and lowest in flying mammals. G content showed the second largest effect (ε^2^ = 0.081), being highest in flying mammals, intermediate in aquatic mammals, and lowest in terrestrial mammals. A content (ε^2^ = 0.038) and C content (ε^2^ = 0.017) were both highest in flying mammals and lowest in aquatic mammals, with terrestrial mammals intermediate; however, for both A and C, the difference between flying and terrestrial mammals was not significant after Bonferroni correction. These compositional differences may be associated with variation in tandem-repeat abundance, lineage-specific mutational biases, and/or differences in selective constraints on *D-loop* sequence composition. However, because nucleotide proportions are compositional and the present composition analysis was conducted at the record level without explicit phylogenetic correction, these compositional patterns should be interpreted as descriptive and hypothesis-generating rather than as definitive evidence of ecological adaptation.

### 4.2. Metabolic Demand and the Elongated D-Loop of Flying Mammals

Powered flight is one of the most energetically demanding forms of locomotion among vertebrates. During flight, bats exhibit metabolic rates 3-5 times higher than those of terrestrial mammals of comparable body size [32], and their mean lifetime energy expenditure is considerably greater than that of non-volant mammals [33]. These elevated metabolic demands are reflected in multiple aspects of bat physiology, including respiratory and cardiovascular specializations and high mitochondrial densities in flight and cardiac muscles [34]. Shen et al. [3] demonstrated that mitochondrial-encoded oxidative phosphorylation (OXPHOS) genes in bats show strong signatures of adaptive evolution, with 23.08% of mitochondrial genes and 4.90% of nuclear-encoded OXPHOS genes exhibiting evidence of positive selection on the ancestral bat lineage. This cytonuclear coevolutionary pattern underscores the critical role of energy metabolism adaptation in the evolutionary origin of flight.

The significantly longer *D-loop* observed in flying mammals may be functionally linked to these heightened metabolic requirements. The *D-loop* contains the promoters for both heavy- and light-strand transcription, and longer *D-loop* regions may harbor additional regulatory elements or tandem repeats that enhance the efficiency of mtDNA replication and transcription initiation [11,31]. Under conditions of sustained high-energy demand, such as those experienced during flight, increased mitochondrial biogenesis and elevated rates of mtDNA replication may be necessary to maintain adequate ATP production. A longer *D-loop* with expanded regulatory capacity might facilitate the rapid and flexible upregulation of mitochondrial gene expression required to meet these energetic challenges.

Several important caveats qualify this adaptive scenario. First, control-region expansion entails potential functional trade-offs that we did not consider in the original formulation of our hypothesis: a longer *D-loop* increases the amount of DNA to be replicated per genome, and selection on replication efficiency has long been hypothesized to favor compact mitochondrial genomes [5,12]. Expanded tandem-repeat arrays can also impose costs, including elevated rates of replication slippage, length instability, and heteroplasmy, and there is no a priori reason to expect that a longer control region improves the accuracy or economy of transcription initiation [12,35]. Second, our dataset contains no functional measurements: we did not characterize *D-loop* secondary structures, promoter architecture, or repeat organization in the sampled taxa, nor did we measure mtDNA copy number, transcriptional output, or respiratory performance. Establishing that longer *D-loops* confer improved regulatory function would require fine-scale structural characterization of control-region sequences combined with direct functional assays (e.g., mtDNA replication and transcription rate measurements, respirometry of flight muscle mitochondria), ideally in a phylogenetically controlled design. Third, because powered flight originated only once in mammals, the “flying mammal” pattern we document is, in effect, the pattern of a single order (Chiroptera). It therefore cannot be statistically separated from other bat-specific traits or from the ancestral state of the chiropteran lineage, and should be regarded as a hypothesis to be tested against additional independent origins of high-metabolism lifestyles rather than as evidence of adaptation.

### 4.3. Aquatic Adaptation and D-Loop Compaction

In contrast to flying mammals, aquatic mammals exhibited the shortest *D-loop* regions and the smallest mitochondrial genomes. Buoyancy in water reduces the energetic costs of supporting body weight during locomotion [35]. Many aquatic mammals, particularly deep-diving cetaceans, experience repeated hypoxic conditions during dives that trigger transient metabolic suppression and reduced oxygen consumption [36]; however, basal metabolic rates in aquatic mammals are not universally lower than those in terrestrial mammals, and some diving species maintain high metabolic capacities during surface intervals. Under such intermittently hypoxic conditions, the requirement for rapid and fine-scale regulation of mtDNA replication and transcription may be reduced relative to that in flying mammals during sustained aerobic flight. We speculate that the shorter *D-loop* observed in aquatic mammals might reflect a more compact regulatory architecture adapted to intermittent hypoxia and the metabolic fluctuations associated with breath-hold diving, rather than to universally lower basal metabolic rates. This hypothesis is speculative, however, and was not supported by our phylogenetically corrected PGLS analyses (*D-loop* overall *p* = 0.463; aquatic vs. terrestrial contrast *p* = 0.252). This interpretation is consistent with the observation that hypoxia-tolerant mammals, including cetaceans, show distinct evolutionary patterns in energy metabolism genes [17,18]. Tian et al. [17] identified positive selection on genes involved in oxygen transport and vasoconstriction regulation in cetaceans, suggesting that aquatic adaptation has shaped multiple aspects of mitochondrial and nuclear genome evolution. Furthermore, cetacean mitochondrial tRNAs exhibit signatures of adaptive evolution [21], and the *D-loop* shows substantial length variation across cetacean species [20], indicating that aquatic environments have influenced both coding and non-coding regions of the mitochondrial genome.

Again, these interpretations must be weighed against the phylogenetic structure of our sample: the aquatic group comprises only two independent transitions to aquatic life (Cetacea and Sirenia). With so few independent evolutionary events, the difference between aquatic and other mammals cannot be statistically attributed to the aquatic lifestyle per se; the compact *D-loop* could equally represent a retained ancestral character state of these two lineages. We therefore present the metabolic and drift-based explanations below as speculative, mutually non-exclusive hypotheses rather than as conclusions. In particular, the intermittent-hypoxia explanation for *D-loop* compaction in aquatic mammals is not supported by our phylogenetically corrected results and should be regarded as a hypothesis for future testing.

Finally, the broad ecological categories used here necessarily mask substantial within-group heterogeneity. Our terrestrial group encompasses fossorial, arboreal, gliding, and semi-aquatic lineages that differ dramatically in metabolic demand, thermoregulatory strategy, and life history; similarly, the aquatic group includes both deep-diving cetaceans that experience prolonged hypoxia and shallow-water sirenians with distinct metabolic profiles. Gliding mammals provide an instructive case for assessing the limitations of such coarse classification: although gliding species were grouped together with other mammals into the terrestrial category, their aerial locomotion represents a distinct functional mode. As an exploratory subgroup analysis, we therefore compared *D-loop* length between gliding mammals and all other mammals, but detected no significant difference (Figure S3). This suggests that the group-level patterns reported here are unlikely to be driven by heterogeneity within this particular subgroup; nonetheless, we caution more broadly that the observed group-level patterns should not be interpreted as evidence that all members of a given ecological category share identical metabolic phenotypes. Rather, these coarse groupings serve as an exploratory framework that captures the dominant locomotor environment while acknowledging considerable ecological and physiological diversity within each group. Future studies employing finer-grained classifications—distinguishing, for example, fossorial, arboreal, gliding, cursorial, semi-aquatic, shallow aquatic, and deep-diving lineages—will be required to isolate the specific selective regimes that may shape mitochondrial genome architecture.

### 4.4. The Role of Genetic Drift in D-Loop Evolution

While metabolic and selective factors likely contribute to *D-loop* length differences, genetic drift may also play a role, particularly in aquatic mammals. The strength of genetic drift is inversely related to effective population size (Ne) [37,38], and many aquatic mammals, particularly cetaceans, have relatively small Ne values due to historical population bottlenecks and current conservation threats [39,40,41]. Small effective population sizes increase the probability of stochastic fixation of neutral or slightly deleterious variants, including insertions and deletions in the *D-loop* region [41,42]. This stochastic process may contribute to *D-loop* shortening in aquatic mammals, although drift alone cannot explain the consistent directional pattern observed across the entire aquatic group. A more comprehensive assessment would require population-genetic modeling of *D-loop* evolution under varying demographic scenarios.

### 4.5. rRNA Gene Length Variation

Unlike the *D-loop*, the *12S* and *16S rRNA* genes exhibited patterns of length variation that were not congruent with differences in complete mitochondrial genome size. The 12S rRNA length ranked as aquatic mammals > flying mammals > terrestrial mammals, whereas the *16S rRNA* length ranked as aquatic mammals > terrestrial mammals > flying mammals. These differences, although statistically significant, were relatively small in absolute magnitude: the maximum difference in median *12S rRNA* length was 18 bp, and the maximum difference in *16S rRNA* length was 12 bp. These values are much smaller than the approximately 420 bp difference in *D-loop* length among groups. However, these statistically significant *p*-values (*12S*: H = 375.56, *p* < 0.001, ε^2^ = 0.22; *16S*: H = 111.97, *p* < 0.001, ε^2^ = 0.07) must be interpreted cautiously because the very large sample size (n = 1674 species) provides extremely high statistical power. The absolute median differences are biologically trivial: *12S rRNA* ranges from 956 bp to 974 bp (maximum difference = 18 bp, ~1.9%), and *16S rRNA* ranges from 1566 bp to 1578 bp (maximum difference = 12 bp, ~0.8%), representing only approximately 1% variation. Ecological group therefore explains only a negligible proportion of the total variance in rRNA gene length. Furthermore, PGLS analysis did not detect a significant association between ecological group and either *12S rRNA* (overall χ^2^ = 4.5, *p* = 0.107; aquatic vs. terrestrial contrast = 11.8 ± 6.2 bp, *p* = 0.059) or *16S rRNA* (overall *p* = 0.703; aquatic vs. terrestrial contrast = 3.7 ± 6.6 bp, *p* = 0.568) length. Taken together, the high statistical significance in uncorrected tests, the minimal biological effect sizes, the negligible proportion of variance explained, and the absence of significant ecological association in the conservative, low-powered PGLS analysis indicate that the observed rRNA length differences among ecological groups should not be interpreted as evidence of adaptive divergence. In addition, the boundaries of the *12S* and *16S rRNA* genes were based solely on unstandardized GenBank annotations.

### 4.6. Phylogenetic Non-Independence, Statistical Power, and the Limits of Our PGLS Analysis

We performed PGLS to examine whether ecological group remained associated with mitochondrial feature lengths after conditioning on shared evolutionary history. The analysis used species-level median values (n = 1674) and a phylogenetic variance–covariance matrix derived from the nested taxonomic hierarchy (order, family, genus) as a proxy for the species tree. Its results must, however, be read in light of several limitations that change their evidential meaning.

First, a rank-based taxonomic tree is not a substitute for a genuine phylogeny. It assumes equal divergence depths across orders, families, and genera and ignores within-genus structure—assumptions that are violated to very different degrees across mammalian clades. Because Pagel’s λ is estimated jointly with the regression for a particular set of branch lengths, the values we report (λ = 0.74 for genome size, λ = 0.51 for the *D-loop*, λ = 0.18 for the rRNA genes) are properties of the proxy matrix as much as of the data, and should not be interpreted as measures of phylogenetic signal in the strict sense.

Second, the ecological predictor is measured with error. Because ecological group was classified at the ordinal level, semi-aquatic lineages (e.g., Hippopotamidae, Lutrinae, Pinnipedia, and Castoridae) and gliding or strongly arboreal taxa are scored as terrestrial. Classical measurement error in a predictor attenuates regression coefficients toward zero, so part of the null PGLS result may reflect noise in the ecological classification rather than the absence of an association.

## 5. Conclusions

This study systematically compared complete mitochondrial genome length and the lengths of the *D-loop*, *12S rRNA*, and *16S rRNA* regions among flying, aquatic, and terrestrial mammals. All four genomic features differed significantly among the three ecological groups. Complete mitochondrial genome length and *D-loop* length showed the same pattern: flying mammals > terrestrial mammals > aquatic mammals. By contrast, rRNA gene lengths followed distinct patterns, with aquatic mammals possessing the longest *12S* and *16S rRNA* genes.

However, PGLS revealed that none of the group-level differences remained significant after accounting for shared evolutionary history. Because flight evolved once and the aquatic lifestyle twice in mammals, these patterns rest on very few independent evolutionary events and largely reflect retained ancestral states rather than derived adaptive shifts. Nevertheless, the concordant patterns observed in the uncorrected comparisons—flying mammals possessing the longest mitochondrial genomes and *D-loops*, terrestrial mammals being intermediate, and aquatic mammals having the shortest—are consistent with the hypothesis that variation in *D-loop* length may be the major contributor to ecological group-specific differences in mammalian mitochondrial genome size. The elongated *D-loop* of flying mammals may reflect adaptation to the high metabolic demands of powered flight, whereas the compact *D-loop* of aquatic mammals may be associated with intermittent hypoxia, reduced metabolic requirements during dives, and genetic drift in small populations. We emphasize that these metabolic and drift-based scenarios are speculative, mutually non-exclusive hypotheses generated from descriptive patterns rather than confirmed adaptive mechanisms. We therefore present our findings as an exploratory, hypothesis-generating comparison. Phylogenetically explicit comparative analyses using a fully resolved species tree, structural characterization of control-region variation, and direct functional assays of replication and transcription efficiency will be required to test the adaptive scenarios proposed here.

## Figures and Tables

**Table 1:** Classification of the three major mammalian ecological groups.

Ecological Group	Order
Flying mammals (Group 1)	Chiroptera
Aquatic mammals (Group 2)	Cetartiodactyla (former Cetacea)
	Sirenia
Terrestrial mammals (Group 3)	Carnivora
	Cetartiodactyla (former Artiodactyla)
	Eulipotyphla
	Lagomorpha
	Macroscelidea
	Monotremata
	Perissodactyla
	Pilosa
	Proboscidea
	Afrosoricida
	Rodentia
	Scandentia
	Dermoptera
	Cingulata
	Dasyuromorphia
	Diprotodontia
	Hyracoidea
	Pholidota
	Primates
	Tubulidentata

**Table 2:** Comparison of mitochondrial *D-loop* nucleotide composition among flying, aquatic, and terrestrial mammals.

Base	Flying Mammals Mean ± SD (%)	Aquatic Mammals Mean ± SD (%)	Terrestrial Mammals Mean ± SD (%)	Kruskal–Wallis H (2)	ε^2^	*p*
A	32.10 ± 2.62	29.91 ± 1.05	31.68 ± 2.44	65.548	0.038	5.84 × 10^−15^
T	26.22 ± 2.70	30.87 ± 1.68	28.88 ± 2.71	185.561	0.110	5.08 ×10^−41^
C	26.07 ± 2.82	24.36 ± 1.69	25.60 ± 3.06	30.116	0.017	2.89 × 10^−7^
G	15.61 ± 2.19	14.86 ± 1.56	13.84 ± 2.02	136.943	0.081	1.83 × 10^−30^

**Table 3:** PGLS analysis of the association between ecological group and mitochondrial genome features (n = 1674 species), in comparison with ordinary least squares (OLS).

Feature	Pagel’s λ	Aquatic β ± SE (*p*)	Flight β ± SE (*p*)	Overall Group Effect, LR Test (*p*)	OLS *p* (Aquatic/Flight)
*D-loop*	0.51	−116.7 ± 101.9 (0.252)	44.9 ± 119.0 (0.706)	1.5 (0.463)	3.8 × 10^−8^/<10^−16^
*12S rRNA*	0.18	11.8 ± 6.2 (0.059)	7.7 ± 7.0 (0.266)	4.5 (0.107)	<10^−16^/9.6 × 10^−14^
*16S rRNA*	0.18	3.7 ± 6.6 (0.568)	−4.1 ± 7.3 (0.574)	0.7 (0.703)	2.4 × 10^−4^/0.15
Complete genome size	0.74	−107.8 ± 104.6 (0.303)	49.6 ± 123.4 (0.688)	1.3 (0.521)	2.3 × 10^−8^/<10^−16^

Note: β coefficients are contrasts relative to terrestrial mammals (reference level); the overall group effect is the likelihood-ratio (LR) test of the two ecological contrasts jointly; Pagel’s λ was estimated by maximum likelihood under the taxonomic proxy tree; OLS *p* values are from equivalent non-phylogenetic regressions on the same species-level medians.

## Data Availability

The mitochondrial genome sequences analysed in this study are publicly available in GenBank (https://www.ncbi.nlm.nih.gov/genbank/, accessed on 2 September 2026). Accession numbers are provided in Supplementary Table S1.

## Supplementary Materials

The following supporting information can be downloaded at: https://www.mdpi.com/article/10.3390/genes17101294/s1, Figure S1: Record-level Kruskal–Wallis test; Figure S2: Kruskal–Wallis test of the random subsampling; Figure S3: Comparison of mitochondrial *D-loop* sequence lengths between gliding mammals and other terrestrial mammals. Table S1: Summary of mitochondrial genome size and lengths of the *D-loop*, *12S rRNA*, and *16S rRNA* regions across 1674 mammalian species; Table S2: Mitochondrial genome features of the three random subsampling datasets for sensitivity analysis; Table S3: Species-level median estimates of complete mitochondrial genome size, *D-loop*, *12S rRNA* and *16S rRNA* lengths across 1674 mammalian species; Table S4: Kruskal–Wallis test results for the complete mitochondrial genome; Table S5: Kruskal–Wallis test results for *D-loop*; Table S6: Kruskal–Wallis test results for *12S rRNA*; Table S7: Kruskal–Wallis test results for *16S rRNA*.

## Abbreviations

The following abbreviations are used in this manuscript:

Abbreviation	Full term
CSB	Conserved sequence blocks
D-loop	Displacement loop
ETAS	Extended termination-associated sequence
HSP	Heavy-strand promoter
LSP	Light-strand promoter
mtDNA	Mitochondrial DNA
Ne	Effective population size
OH	Origin of heavy-strand replication
OXPHOS	Oxidative phosphorylation
PGLS	Phylogenetic generalized least squares
ROS	Reactive oxygen species
TAS	Termination-associated sequence
