# Supplementary material for: Mitochondrial Genome Size and D-Loop Variation in Flying, Aquatic, and Terrestrial Mammals: An Exploratory Comparative Analysis
Source: Genes (Basel). 2026 Oct 16;17(10):1294. doi: 10.3390/genes17101294 (PMC13704294; doi:10.3390/genes17101294)
Supplement: Supplementary file 1 [file genes-17-01294-s001.zip › Table S7.pdf]

Table S7 Kruskal-Wallis test results for *16S rRNA*

Group (Median)	Sequence size
1 (n = 196)	1,566
2 (n = 77)	1,578
3 (n = 1,401)	1,570
Kruskal-Wallis test statistic H	111.97

* $p<0.05$ ** $p<0.01$
