# Supplementary material for: Mitochondrial Genome Size and D-Loop Variation in Flying, Aquatic, and Terrestrial Mammals: An Exploratory Comparative Analysis
Source: Genes (Basel). 2026 Oct 16;17(10):1294. doi: 10.3390/genes17101294 (PMC13704294; doi:10.3390/genes17101294)
Supplement: Supplementary file 1 [file genes-17-01294-s001.zip › Table S4.pdf]

**Table S4** Kruskal-Wallis test results for the complete mitochondrial genome

| Group (Median) | Sequence size |
|---|---|
| 1 (n = 196) | 16,740 |
| 2 (n = 77) | 16,386 |
| 3 (n = 1,401) | 16,544 |
| Kruskal-Wallis test statistic H | 177.44 |

* $p<0.05$ ** $p<0.01$
