# Supplementary material for: Mitochondrial Genome Size and D-Loop Variation in Flying, Aquatic, and Terrestrial Mammals: An Exploratory Comparative Analysis
Source: Genes (Basel). 2026 Oct 16;17(10):1294. doi: 10.3390/genes17101294 (PMC13704294; doi:10.3390/genes17101294)
Supplement: Supplementary file 1 [file genes-17-01294-s001.zip › Table S5.pdf]

Table S5 Kruskal-Wallis test results for *D-loop*

Group (Median)	Sequence size
1 (n = 196)	1,284
2 (n = 77)	914
3 (n = 1,401)	1,069
Kruskal-Wallis test statistic H	181.47

* p<0.05 ** p<0.01
